# Reassessing Regional Tick-Borne Encephalitis Endemicity in Poland Through Seroprevalence Analysis in Blood Donors, 2021–2022

**DOI:** 10.3390/pathogens15070720

**Published:** 2026-07-08

**Authors:** Katarzyna W. Pancer, Magdalena Rosińska, Gerhard Dobler, Daniel Rabczenko, Agnieszka Kołakowska-Kulesza, Beata Gad, Anna Poznańska, Piotr Grabarczyk

**Affiliations:** 1 National Institute of Public Health NIH-NRI, Chocimska 24, 00-791 Warsaw, Poland; mrosinska@pzh.gov.pl (M.R.); drabczenko@pzh.gov.pl (D.R.); bgad@pzh.gov.pl (B.G.); apoznanska@pzh.gov.pl (A.P.); 2 Bundeswehr Institute of Microbiology, 80937 Munich, Germany; gerharddobler@msn.com; 3 Department of Virology, Institute of Heamatology and Transfusion Medicine, 00-791 Warsaw, Poland; pgrabarczyk@ihit.waw.pl

**Keywords:** TBE, TBEV, surveillance, blood donors, Poland, infection-induced antibodies, vaccine-induced antibodies

## Abstract

TBEV is a major cause of viral central nervous system infections in Europe, with heterogeneous geographical distribution and substantial underdiagnosis in low-incidence regions. This study aimed to evaluate the validity of regional TBE risk classification in Poland by combining surveillance-based incidence data with serological markers of TBEV exposure. Plasma samples from 5541 blood donors residing in nine regions were tested by anti-TBEV IgG ELISA, followed by confirmatory VNT, IFA, and anti-NS1 IgG ELISA to differentiate infection-induced from vaccine-induced antibodies. Regions were classified based on average TBE incidence registered in surveillance systems in 2015–2019. Overall, 272 (4.9%) donors were positive in TBEV IgG ELISA and 177 (3.2%) also in a confirmatory assay. Seroprevalence expressed by markers consistent with past TBEV infection (anti-NS1 IgG) was estimated at 0.13% (95% CI 0.05–0.26%), ranging from 0.0% to 0.33% by voivodeship, whereas vaccine-induced immunity accounted for the majority of samples with detected specific antibodies (3.1%). Surprisingly, seroprevalence in a highly affected region (0.29%) was at the same level as in one that was less affected (0.33%). Moreover, all except one seropositive donor lived in urban areas. In four out of nine voivodeships, no anti-NS1 TBEV IgG was detected. By utilising precise laboratory algorithms, we demonstrated a much lower seroprevalence that estimated from prior research relying on screening tests. In addition, we confirmed low vaccination coverage. Integrating sero-epidemiological data with surveillance systems may improve risk assessment and inform targeted prevention strategies.

## 1. Introduction

Tick-borne encephalitis virus (TBEV) is the leading cause of viral encephalitis (tick-borne encephalitis, TBE) and meningoencephalitis in the Polish population. The primary route of TBEV transmission to humans is through tick bites. Given that, as in many other European countries, Poland has experienced a recent increase in tick-borne diseases [1], a corresponding rise in the number of TBEV infections and human TBE cases can be anticipated. Occasional outbreaks have also been linked to the consumption of unpasteurized milk from infected sheep or goats [2,3]. In addition, TBEV transmission via transplanted organs has been documented in Poland [4], and sporadic cases of this transmission route have been reported in other countries [5,6].

The geographical distribution of TBEV-infected ticks in Poland is heterogeneous. Certain regions, particularly in the northeastern part of the country, have consistently reported the presence of TBEV-positive ticks, whereas other areas, notably in the northwest, have repeatedly tested negative [7]. However, no clear correlation has been observed between the regional distribution of TBEV in ticks and the incidence of reported TBE cases in humans [8,9]. Moreover, TBEV RNA has been detected in ticks collected from regions with low reported TBE incidence [8,10].

Most TBEV infections are asymptomatic or present with non-specific, mild symptoms; however, in approximately 10–15% of cases, central nervous system involvement occurs, often leading to long-term neurological sequelae. Diagnosis relies on laboratory testing; nevertheless, in regions where specific diagnostic assays are not routinely available, symptomatic TBE cases may remain undetected [11,12], as has been reported in several areas of Poland [8,13]. Vaccination remains the most effective strategy for the prevention of TBE [14,15,16,17].

In Poland, vaccination against TBE is recommended; however, the vaccine is not reimbursed through the public healthcare system. Although exact estimates for vaccine coverage are not available, it is considered to be very low [18], with 0.15% to 0.23% of the population having received a dose of vaccine annually in 2019–2022 [19]. Vaccination most often occurs in occupational settings. According to the current Immunization Program [20], vaccination is recommended for forest workers, soldiers, firefighters, border guards, and farmers. For individuals at occupational risk in highly affected areas, vaccination costs should be covered by employers in accordance with the Labour Code (Article 222) [21]. Nevertheless, the definition of “highly affected” areas remains ambiguous and allows for subjective interpretation. In contrast, several other countries have adopted more clearly defined and operational vaccination criteria [15]. The application of these recommendations could follow the World Health Organization (WHO) guidance. The WHO recommends the implementation of vaccination in regions where the annual TBE incidence is ≥5 cases per 100,000 inhabitants. Based on national surveillance data, by 2023, only one region in Poland—Podlaskie voivodeship, in the northeastern part of the country—met this criterion. In all other voivodeships, except Warmińsko-Mazurskie voivodeship, reported TBE incidence ranges from 0.0 to 1.0 per 100,000 inhabitants [7,22,23].

However, limited regional access to diagnostic testing may lead to underdiagnosis, calling into question the reliability of regional classification based solely on registered incidence rates, and additional data, such as seroprevalence are needed to confirm it. Although seroprevalence studies were already conducted in Poland, we note that those studies [8,9,23,24,25] did not distinguish between vaccine-induced and post infection-acquired antibodies. Given increasing and regionally heterogeneous vaccination coverage, this limitation may bias interpretations of seroprevalence data and obscure the true epidemiological situation.

The aim of the presented study was to verify current classification of defined areas in Poland based on epidemiological surveillance data as high, moderate, and less affected with data from serological TBEV marker screening in blood donors, specifically taking into account the prevalence of vaccine-induced antibodies. In addition, we aim to identify the seroprevalence by demographic group to understand population immunity levels.

## 2. Materials and Methods

### 2.1. Selection of Voivodeships

Due to the relatively small number of reported TBE cases and year-to-year variability in incidence, surveillance data were analyzed over a 5-year period. Based on the mean annual incidence over 5 years (number of reported TBE cases per 100,000 population), voivodeships were classified as highly (≥5.0), moderately (≥0.1 to <1.0), or lowly affected (<0.1). Only one voivodeship in Poland met the criteria for an area of high exposure to the TBEV virus, whilst the remaining 15 were below this threshold (>5.0 per 100,000 population) set by the World Health Organization (WHO). In the voivodeship with the second-highest TBE incidence, Warmińsko-mazurskie voivodeship, the TBE incidence was 3.30 in 2019, which differed significantly from the values recorded in the rest of the 14 voivodeships (with TBE incidence < 1 per 100,000). Moreover, the 5-year average TBE incidence was also below 1/100,000 in those 14 voivodeships. For this reason, for the purposes of this study, two additional criteria were introduced to better reflect the TBE epidemiological situation in Poland, resulting in the division of voivodeships into three exposure categories.

**Figure 1:**
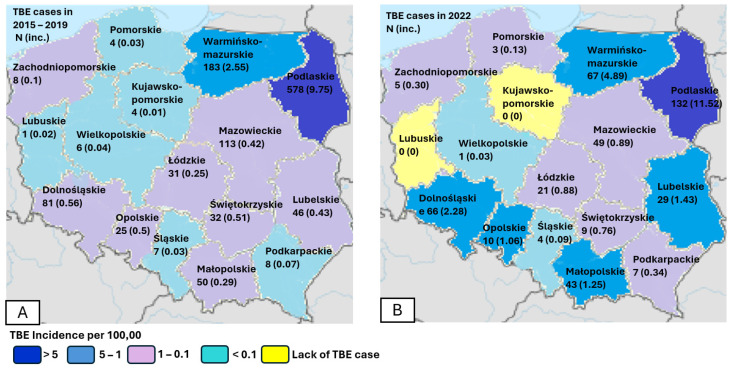
Epidemiological situation of TBE in Poland based on epidemiological surveillance data: (**A**). Number of TBE cases (incidence) reported in Poland in 2015–2019 by voivodeship; (**B**). Number of TBE cases (incidence) reported in Poland in 2022 by voivodeship.

Using national surveillance data from 2015–2019 (Table A1), voivodeships were classified as follows (Figure 1):

- Highly affected: Podlaskie, (>5.0 per 100,000 population);

The 14 voivodeships characterized by TBE incidence < 1 per 100,000 can be divided into two groups:

- Moderately affected: Dolnośląskie, Łódzkie, Mazowieckie, Lubelskie, Opolskie, Małopolskie, and Świętokrzyskie. In these regions, with a 5-year TBE incidence ranging from 0.1 to <1.0 per 100,000 population, TBE cases were reported annually, and the virus was sporadically detected in environmental studies [7]. One possible explanation was the presence of smaller administrative units within these voivodeships with higher local incidence despite an overall voivodeship-level incidence above 1 per 100,000.
- Low affected: Zachodniopomorskie, Pomorskie, Kujawsko-Pomorskie, Lubuskie, Podkarpackie, Śląskie, and Wielkopolskie. In these regions, only sporadic TBE cases were reported, with a mean 5-year incidence below 0.1 per 100,000 population; many of these cases are likely associated with travel to more highly affected areas.

### 2.2. Study Population and Design

The plasma bank was established in 2021–2022 as part of the project “TBE seroprevalence in Poland” (TBEseroPL), conducted at the National Institute of Public Health–National Institute of Hygiene–National Research Institute (NIPH NIH–NRI). The study protocol was approved by the Ethics Committee of the NIPH NIH–NRI (approval no. 13/2021, 19 November 2021).

The sampling frame comprised blood donors registered at Regional Blood Transfusion Centers (RBTCs) located in nine of the sixteen Polish voivodeships: Dolnośląskie, Kujawsko-Pomorskie, Lubelskie, Łódzkie, Małopolskie, Podkarpackie, Podlaskie, Pomorskie, and Zachodniopomorskie. The necessity of selection was dictated by feasibility considerations, and the voivodships were purposefully selected to represent geographically different regions, which in addition fell under all the three categories for the TBE mentioned above. Each RBTC provided residual EDTA plasma samples from randomly selected adult donors (≥18 years) collected during routine mandatory screening for the hepatitis C virus (HCV), hepatitis B virus (HBV), human immunodeficiency virus (HIV), and *Treponema pallidum*. All plasma samples were transported on dry ice and stored at −20 °C until analysis.

### 2.3. Sample Size Calculation

Sample size was determined using a precision-based sample-size calculation in STATA (version 18.0 SE; StataCorp LLC, College Station, TX, USA), assuming significance level (α) of 0.05 and a statistical power of 0.80. Calculations were performed using the normal approximation to the binomial distribution, two-sided confidence interval, and were conducted separately for highly affected, moderately affected, and low-affected areas.

A previous seroprevalence study by Stefanoff et al. [25], based on samples collected between 1996 and 2005, reported an anti-TBEV IgG seroprevalence of 4.3% in the endemic Podlaskie voivodeship using ELISA screening. The samples were taken from people seeking medical advice for conditions other than neurological disorders.

The majority of positive samples (7/10) were confirmed as infection-induced by indirect immunofluorescence (IIF) and hemagglutination inhibition (HI) assays. In contrast, in non-endemic regions, the proportion of confirmed TBEV infections among ELISA-positive samples was lower (4/14 retested sera), indicating that similar ELISA seroprevalence rates may correspond to substantially different proportions of true infections.

Given evidence of TBE incidence in several moderate- and low-affected areas that may remain undetected due to limited diagnostic testing, we assumed identical expected seroprevalence for moderately and low-affected regions for the purposes of sample-size estimation.

The following assumptions were applied:

- Highly affected areas: expected seroprevalence of 4%, with an acceptable confidence interval width of 3% (e.g., 2.5–5.5%), resulting in an estimated sample size of 688.
- Moderately and low-affected areas: expected seroprevalence of 1.5%, with an acceptable confidence interval width of 1% (e.g., 1.0–2.0%), resulting in an estimated sample size of 2346.

Accordingly, target sample sizes were set at approximately 700 samples for highly affected areas and 2400 samples for both moderately and low-affected areas. The study included one highly affected voivodeship (Podlaskie), four moderately affected voivodeships (Dolnośląskie, Lubelskie, Łódzkie, and Małopolskie), and four low-affected voivodeships (Kujawsko-Pomorskie, Podkarpackie, Pomorskie, and Zachodniopomorskie), with an equal allocation of approximately 600 samples per voivodeship.

### 2.4. Screening Assays

As enzyme-linked immunosorbent assays (ELISAs) are the primary methods used for serological diagnosis of TBE, a commercially available anti-TBEV ELISA assay marketed in Poland was selected. All plasma samples were tested using the Euroimmun anti-TBEV IgG ELISA (Anti-TBEV ELISA IgG, EI 2661-9601 G; Euroimmun, Lübeck, Germany) according to the manufacturer’s instructions. Results were expressed as the ratio of the optical density (OD) of the sample to that of the calibrator, with values > 1.1 interpreted as positive, <0.8 as negative, and 0.8–1.09 as borderline. The sensitivity of this test was evaluated by Reusken et al. [26] as 83% but with a specificity >60%.

### 2.5. Confirmatory Assays

Confirmatory testing was performed at the Bundeswehr Institute of Microbiology (Munich, Germany). Virus neutralization tests (VNTs) were conducted to verify the specificity of ELISA-positive results. To differentiate between vaccine-induced and infection-induced antibodies, an anti-NS1 IgG assay was applied as previously described [27].

Upon receipt, all selected sera were re-tested using the same ELISA screening assay to confirm reactivity but in an automatic system. This step was necessary due to repeated freeze–thaw cycles, shipment conditions, and limited sample volumes, which may affect antibody stability.

Criteria for confirmatory testing included sera with repeated positive results (ratio ≥ 1.1), borderline results (ratio 0.8–1.09 R), and negative samples with the result 0.7–0.8 R or a marked decrease in antibody levels between tests. In total, 278/5541 sera met these criteria and were subjected to confirmatory analysis using the following methods:

- Detection of anti-NS1 TBEV IgG by an in-house ELISA performed according to published protocols [27,28]. As NS1 is a non-structural protein expressed only during viral replication, the presence of anti-NS1 antibodies indicates natural TBEV infection. This ELISA assay has demonstrated a sensitivity of >94% and a specificity of >93% in broadly cross-reacting sera from patients with vaccinations against flaviviral diseases and single or multiple flavivirus infections, respectively [27].
- Virus neutralization test (VNT) was conducted according to established protocols, with an NT_50_ threshold > 1:10 considered confirmatory [27,28], to verify ELISA specificity and exclude cross-reactivity with other flaviviruses.
- Indirect immunofluorescence test (IIFT) was performed following published procedures [27,28] as a complementary method to identify cross-reactive antibodies against other flaviviruses.

Confirmatory analyses were not performed in 34 samples due to insufficient sample volume.

**Figure 2:**
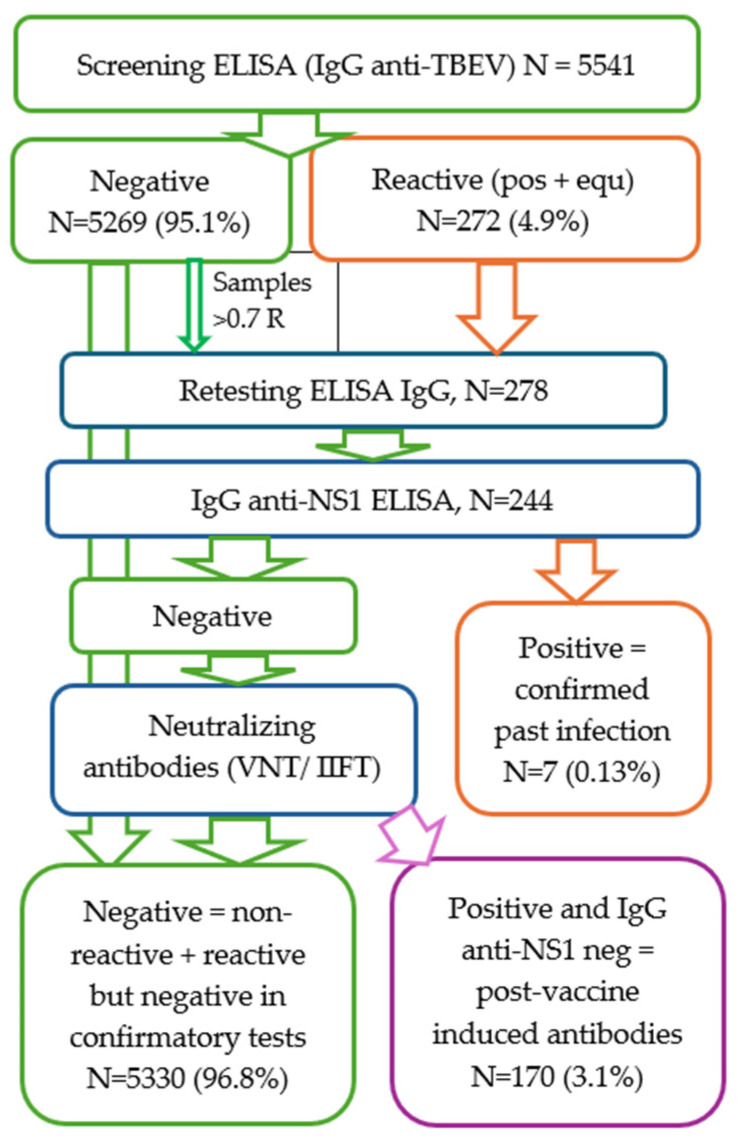
Algorithm of the study.

All assays were performed according to the diagnostic algorithm presented in Figure 2.

As seroprevalence is the proportion of a population who test positive for a specific disease, particularly antibodies to an infectious agent, in their blood serum or plasma, the specificity of the antibodies must be verified. The first stage in verifying the specificity of the detected IgG antibodies was to determine whether the sample contained antibodies capable of neutralising the TBEV virus by testing it using VNT and IIFA methods. At this stage, we observed seropositivity, i.e., the co-occurrence of antibodies produced in response to vaccination against tick-borne encephalitis and antibodies produced in response to natural viral infection. The next stage was to determine seroprevalence, i.e., the detection of antibodies induced exclusively by TBE virus infection. This was performed by detecting the presence of IgG antibodies against the NS1 protein of the TBEV virus.

### 2.6. Statistical Analysis

Univariable logistic regression analyses were performed to assess associations between selected factors and TBEV seropositivity (binary outcome: presence vs. absence of confirmed antibodies). Results are presented as odds ratios (ORs) with corresponding 95% confidence intervals (CIs) and *p*-values. Seroprevalence estimates were calculated as proportions with 95% confidence intervals, and differences between groups were assessed using appropriate statistical tests. A two-sided significance level of α = 0.05 was applied. Statistical analyses were conducted using R software version 3.4.1 [29].

## 3. Results

We analyzed results from 5541 blood donor samples; donor age ranged from 18 to 66 years. Among the participants, 52.3% were ≤40 years old and 47.7% were older than 40 years. The median age was 40.0 years (interquartile range [IQR]: 30.0–46.0 years). The study population comprised 2370/5541 women (42.8%) and 3171/5541 men (57.2%). The majority of donors resided in urban areas 3372/5541 (60.9%) (Table A2).

Reactivity detected in the screening ELISA test (positive and equivocal anti-TBEV IgG results) was found in 272/5541 samples (4.9%). The distribution of reactivity differed significantly between voivodeships (ranged 2.0–9.5%), and difference of reactivity between less and highly affected regions was more than five-fold (*p* < 0.001) (Table A3). The highest value of reactivity (9.5%, 67/703) was observed in the voivodeship previously classified as highly affected (Podlaskie). Notably, an elevated reactivity level was also detected in voivodeships classified as low affected: Kujawsko-Pomorskie (8.2%, 49/600) and Zachodniopomorskie (5.3%, 32/600).

A significant association between sex and reactivity was identified (*p* < 0.001). Overall, the odds of reactivity in anti-TBEV antibodies were 1.9 times higher in men than in women (OR = 1.86; 95% CI: 1.43–2.43; *p* < 0.001) (Table A3). No significant difference in anti-TBEV IgG reactivity was observed with respect to the age group (OR = 0.86; 95% CI: 0.67, 1.09; *p* > 0.05) and place of residence: 4.6% (99/2170) among rural residents and 5.04% (170/3371) among urban residents (OR = 0.92; 95% CI: 0.72, 1.18; *p* > 0.05). It should be noted that most previous studies on TBEV seroprevalence in Poland ended at this stage.

### 3.1. Determination of TBEV Seroprevalence

Based on screening and confirmatory testing, immunological status was determined for 5507 blood donors. Evidence of past TBEV infection was identified in seven individuals (0.13%) only, while neutralizing antibodies without IgG anti-NS1 TBEV were detected in 170 individuals (3.1%, 170/5507), and no detectable TBEV antibodies in 5330 individuals (96.8%, 5330/5507) (Table 1).

Seroprevalence (expressed as the IgG antibodies against NS1 TBEV) was 0.13%, and the antibodies were detected in seven blood donors from five out of nine voivodeships. The characteristics of these blood donors are presented in Table 1. Seroprevalence varied by regions: unexpectedly, the highest was determined in Pomorskie voivodeship—classified as less affected (0.33%, 2/598), followed by highly affected Podlaskie voivodeship (0.29%, 2/698); next, Dolnośląskie, Małopolskie, and Podkarpackie voivodeships (respectively, 0.17%, 0.17%, 0.16%). No anti-NS1 TBEV IgG antibodies were detected in four voivodeships classified as less or moderately affected (Table 1).

Anti-TBEV NS1 IgG was detected in four men and three women. Surprisingly, all but one person (from the Pomorskie voivodeship) lived in urban areas (Table 1). Due to the very low seroprevalence rates in the examined voivodeships, it was not possible to carry out a more detailed analysis.

### 3.2. Vaccination-Induced Antibody Prevalence

Vaccination-induced immune responses were identified in 170/5707 (3.1%) donors, predominantly among men—73.5% (125/170). This male predominance was observed across all analyzed regions. However, vaccination-induced neutralizing antibodies frequency distribution varied between voivodeships, ranging from 1.7% in Dolnośląskie (10/603) to 7.4% (52/698) in Podlaskie, with the highest proportion observed in the highly affected region. In some voivodeships (e.g., Łódzkie), vaccination-induced antbodies were found exclusively among men. Among blood donors who had received a vaccine, the majority were city dwellers (61.2%, or 104 out of 170) (Table 2).

### 3.3. Results by Region Classification

Given the very low seroprevalence based on sporadic detection of anti-NS1 IgG across voivodeships, additional analyses were performed by aggregating data from highly affected regions, moderately affected regions (four voivodeships combined), and lowly affected regions (four voivodeships combined) (Table 3).

Table 3 presents aggregated serological results according to regional TBE incidence classification. Screening reactivity was highest in highly affected region, where it reached 9.6% (67/698), compared with 3.1% (75/2404), in moderately affected regions and 5.4% (130/2405) in less affected regions. Confirmed TBEV infection remained rare in all regional categories: 0.29% in highly affected regions, 0.08% in moderately affected regions, and 0.12% in less affected regions. Post-vaccination reactivity was also highest in highly affected regions, reaching 7.4% (52/698), compared with 2.2% and 2.7% in moderately and less affected regions, respectively.

## 4. Discussion

A study conducted to verify the assessment of TBEV risk in Poland demonstrated lack of significant difference in seroprevalence between regions classified as having low or moderate exposure or high exposure based on epidemiological surveillance data. The seroprevalence was unexpectedly low making our study most likely underpowered to detect such differences. The study also confirmed the low level of TBE vaccination coverage in Poland. In previous studies conducted in Poland, the total level of antibodies specific to the TBEV virus, resulting from infection with the virus and vaccination together, was referred to as seroprevalence. However, it was actually seropositivity rather than seroprevalence. In our studies, we were able to distinguish between seroprevalence, which indicates exposure to TBEV infection (IgG anti-NS1 TBEV positive), and the prevalence of post-vaccination immune responses. Our study was the first attempt to understand TBE epidemiology in Poland using seroprevalence approach based on differentiation of vaccine-induced and infection-induced antibodies. The higher seroprevalence attributable to infection was observed in the region identified as less-affected region (Pomorskie), at the same level as in highly affected in surveillance data. Both voivodeships are located in Northern Poland. However, because only a small number of blood donors were identified as having antibodies indicative of previous infection, a comprehensive analysis of the results was not possible.

Previous studies on TBEV seroprevalence in Poland have been conducted mainly among groups other than blood donors (healthcare workers, individuals seeking medical care for reasons other than viral infections of the nervous system, etc.), which may also have resulted in seroprevalence estimates that were lower than expected.

Due to changes in the epidemiology of tick-borne encephalitis virus (TBEV) infections and the increasing number of detected and reported cases, tick-borne encephalitis (TBE) has become a major public health concern in Europe. Consequently, many countries have conducted seroprevalence studies to estimate the immune status of their populations resulting from both natural TBEV infection and vaccination, including Romania, Slovenia, Germany, Poland, the Czech Republic, Italy, Denmark, Sweden, and Serbia [27,28,30,31,32,33,34,35,36,37,38,39,40,41]. More refined testing strategies that differentiate between immunity acquired through past infection and vaccination allow for a more accurate estimation of infection incidence and population susceptibility. Our study represents one of such enhanced approaches [27,28,37,38]. Despite several limitations discussed below, the use of blood donor samples in seroprevalence studies enables comparisons between countries. In most European countries, blood donation is voluntary and non-remunerated, and similar pre-donation screening procedures are applied, which supports the reliability and comparability of results. Comparable studies have recently been conducted in Romania, Germany, and Switzerland [28,31,32,37,39].

In our study conducted among Polish blood donors, the overall proportion of reactive samples (with positive or borderline anti-TBEV IgG ELISA screening results) was 4.9%. The substantial regional differences were observed between voivodeships, ranging from 2.0% to 9.5%. The reactivity frequency obtained is consistent with the results obtained by other researchers in Poland or other countries [32,41] in previous years, who did not apply further laboratory tests. In accordance with current research models, we continued our investigations to determine seroprevalence based on the results of tests confirming the presence of specific antibodies against TBEV. The seropositivity rate determined in this way was 3.2% for the entire study group. Significant variation was still observed between voivodeships, and the highest value was still observed in the Podlaskie.

Seroprevalence was referred to as the proportion of the population with a history of confirmed infection, identified in our study was low (0.13%, 7/5507), significantly lower than the reactivity (4.9%). A similar discrepancy between reactivity and seroprevalence was also observed in Romania (reactivity 3.2% and seroprevalence 0.62%, respectively); but in their case, one highly affected region noted seroprevalence of 9.7% [32]. In our study, seroprevalence remained low even in regions considered to have high exposure (seroprevalence 0.29%, 2/698), highlighting the need for further investigation. One possible explanation is that blood donors residing in high-risk areas may adopt preventive measures, including vaccination, due to their awareness of the potential risk of transmitting infection to blood product recipients, or may wear appropriate clothing and use repellents. It is also possible that highly affected remote communities donate blood less frequently and we, therefore, were not able to capture them.

In contrast, reactivity reported in Germany were markedly higher: positive IgG results were detected in 57% of 2220 blood donors [28,37]. Importantly, that study focused on donors from a highly TBE-endemic region in Southern Germany, where anti-TBE vaccination is highly recommended. In our study, although the overall seropositivity rate was 3.2% among 5507 donors, the highest regional prevalence of positive confirmation results—observed in a voivodeship classified as highly affected—reached 7.7% (54/698). A comparison of seropositivity rate in the region with high incidence rates (according to the WHO definition) in Poland (Podlaskie) and Germany shows significant differences, which may be the result of fewer infections and a likely lower vaccination rate in Poland. However, such significant discrepancies in the results from two different “highly affected” regions warrant further analysis. Data on TBE vaccination coverage in Poland are limited, as reporting of TBE vaccination is not mandatory. Estimates are therefore based on vaccine sales rather than medical records. Despite the absence of a national vaccination registry, an increasing trend in vaccination uptake has been noted in recent years. According to the report *Tick-borne encephalitis in Poland and worldwide. Assessment of the epidemiological situation of TBE in Poland* (28 February 2021) [18], although vaccination coverage increased nearly threefold between 2015 and 2019, overall coverage remained low, at approximately 2.5–4% of the Polish population.

The low vaccine coverage in Poland is consistent with our result of only 3.1% individuals having serological profile consistent with previous immunization. In a study conducted by Bojkiewicz et al. in 2021 in the Podlaskie [24], the authors analyzed the seroprevalence of tick-borne encephalitis virus in two cohorts: 298 blood donors and 180 children (aged 2–17) hospitalized for viral gastroenteritis. The attitudes of adult donors and the parents of hospitalised children towards vaccination against tick-borne encephalitis were also assessed. It should be noted that this study was conducted in the region with the highest risk of TBE infection in Poland, which is likely to be the region where residents have the highest level of general knowledge about TBE. Based on survey data, only 13% of blood donors had been vaccinated against TBE prior to the study, with higher vaccination rates among men (15%) than women (7%), consistent with our findings. Vaccination coverage among parents of hospitalized children was even lower (9%), and none of the children had been vaccinated.

According to Polish national surveillance data, most TBE cases occur among unvaccinated individuals. Assessment of TBEV seroprevalence among unvaccinated individuals is particularly useful for estimating the frequency of asymptomatic infections. In the study by Bojkiewicz et al. [24], reactivity of IgG against TBEV was found in 5% of unvaccinated adults and 2% of children. These data, as well as results reported by Dobler et al. [28], indicate that asymptomatic or mild TBEV infections occur relatively frequently, unlike our results indicating a much lower seroprevalence. Of note, the previous studies did not include the confirmation assay step, which could at least partly explain the difference. The question remains whether antibodies produced during asymptomatic TBEV infection are as persistent as those produced during symptomatic infection, or whether they disappear more quickly.

Detection of anti-NS1 TBEV antibodies indicates immune responses to virus infection, typically acquired through environmental exposure or consumption of unpasteurized dairy products.

Overall, the seroprevalence (anti-NS1 TBEV IgG) was unexpectedly low even in highly affected regions (0.13% overall and 0.29% in Podlaskie voivodeship) in our study. Moreover, the seroprevalence determined in Pomorskie voivodeship, classified as a low-affected area, was at the same level as in highly affected voivodeship (respectively, 0.33% and 0.29%). Overall, in five out of nine voivodeships, seroprevalence (based on confirmed infection) was >0. The low seroprevalence precluded meaningful comparisons between the regions, as we assumed much higher values for the sample-size estimation. Notably, the estimate was significantly lower than studies in Southwestern Germany using similar protocols, and all confirmatory testing was performed in the same reference laboratory in Munich, Germany [5,27,28,31,38,39,42] despite the vaccination coverage being significantly lower in Poland.

Also, the overall rate of detected and confirmed anti-TBEV antibodies in our study (3.2%) was lower than anticipated based on the literature data. Studies conducted in blood donors in Sweden (2018–2019) and in highly endemic regions of Southern Germany reported substantially higher seropositivity rates: respectively: from 9.7% to 64% and from 55% to 84.8%, depending on the studies [27,38,42]. Also, a study in the general population in the Czech Republic (2001) indicated higher value (26.3%) than in our study [35]. The values obtained in our study are rather similar to the seropositivity rate in Denmark [40]. A hypothesis that there is a difference between the expected and determined seropositivity rate due to lower vaccination rates or lower risk in Poland than in neighboring countries needs detailed verification.

Bojkiewicz et al. [24] also assessed previous confirmed TBEV infections and vaccination responses among adults. Notably, four individuals with a documented history of TBE were seronegative at the time of testing, and only 68% of vaccinated individuals were anti-TBEV IgG positive. In contrast to these results, other studies conducted in Europe [42,43,44] indicate a rather stable response, lasting up to 15 years, after vaccination. These findings highlight a key limitation of seroprevalence studies: protective antibody levels depend on time since vaccination or infection, individual health status and age, antibody persistence, and the presence of so-called natural boosters resulting from environmental exposure.

Furthermore, still insufficient information is available regarding the duration of anti-NS1 antibody persistence and potential differences between asymptomatic infections and cases with neurological symptoms. Research conducted in Latvia indicates that anti-NS1 antibodies are also detected in vaccinated individuals, probably as a result of asymptomatic or mild infections that remain unrecognized [45]. Girl et al.’s [42] study indicated unexpected high prevalence (>97%) of asymptomatic cases in highly affected areas and with high level of anti-TBE vaccination coverage area in Germany. Repeated seroprevalence studies conducted at multi-year intervals could substantially improve understanding of TBE distribution, exposure levels, and inform public health strategies, including vaccination campaigns.

Interestingly, six of seven individuals with detectable anti-NS1 TBEV IgG resided in urban areas. This contrasts with surveillance analyses [18], which identify rural residence and prolonged exposure to forested or agricultural environments as major risk factors. One possible explanation is that urban residents—potentially tourists—may be exposed due to inadequate preventive measures, whereas repeated low-level exposure among rural residents may act as natural immune boosters. Another possible explanation is that, because blood donation centers are primarily located in large urban areas, many blood donors may reside either in these cities or in nearby suburban regions. This may account for the higher proportion of individuals with a history of confirmed infection living in urban areas compared with rural populations. This hypothesis warrants further investigation.

High proportions of anti-TBEV IgG-reactive blood donors were also observed in Kujawsko-Pomorskie and Zachodniopomorskie voivodeships (8.2% and 5.3%, respectively), which are regions classified as low-incidence areas based on surveillance data. The discrepancy between the reactivity rate and the seropositivity rate identified in these provinces (8.2% and 5.3% vs. 2.5% and 3.3%, respectively) indicates a substantial proportion of cross-reactive or non-specific responses in the screening ELISA assay in these regions. No markers of recent or past infections were detected in these regions in our study. One possible explanation is the presence of military training grounds, with a higher proportion of vaccinated against other flaviviruses. Although establishment of other flaviviruses in these regions is not documented, it cannot be excluded. Confirmation would require virus neutralization tests to assess potential cross-reactivity, which is well documented among flaviviruses [43,44,46].

Markers of past TBEV infection (anti-NS1 IgG) were detected in donors, not only from highly affected voivodeship but also from Dolnośląskie (1), Małopolskie (1), Podkarpackie (1), and Pomorskie (2) voivodeships. While the first two regions are classified as moderately affected, the latter two are considered low-incidence areas. This may reflect domestic travel patterns, particularly intensified during the COVID-19 pandemic, or underreporting of TBE cases in these regions. As shown by Paradowska-Stankiewicz et al. (2023) [18], comparison of surveillance data with Nationwide General Hospital Morbidity Study (NGHMS) records revealed temporary (during COVID-19 pandemic) substantial underestimation of TBE incidence, confirmed as a significant discrepancy between the number of TBE cases reported to the surveillance system and the number of hospitalizations due to TBE during the same period and in the same region, particularly in Podkarpackie and Pomorskie voivodeships.

This conclusion is supported by the study of Zajkowska et al. (2025) [13], who demonstrated that limited diagnostic capacity was one of significant factors contributing to underreporting of TBE. By retrospectively testing patients initially diagnosed with other viral neurological conditions, they identified 124 previously unreported TBE cases among 766 patients across 10 voivodeships. Similar studies conducted in Sweden detected only one such case (1/137) [47]. Our study, while not negating these findings, does not provide conclusive data on possible re-assignment of risk class for moderate- and low-risk regions, due to overall low seroprevalence.

## 5. Conclusions

Our results suggest low exposure to TBEV infection across regions in Poland. However, although not a significant difference, the highest risk of infection remains in the most affected regions of Poland, particularly the Podlaskie voivodeship. At the same time, some regions with lower reported incidence, such as the Pomorskie voivodeship in Northern Poland could be in fact more affected, but a study with a larger sample size than ours would be necessary to confirm it.

Our research confirms the low TBE vaccination coverage in Poland. Furthermore, it indicates that previous TBEV seroprevalence studies conducted in Poland may largely reflect the presence of post-vaccination antibodies not exposure to infection. This requires further research and analysis.

## Figures and Tables

**Table 1:** TBEV seroprevalence by nine voivodeships in Poland and characteristics of positive blood-donors.

Voivodeship	Regional TBE Incidence Classification	N	Confirmed TBEV Infection = Seroprevalence % (n/N)	Exact 95% CI for Seroprevalence	Characteristics of Blood Donors with Confirmed TBEV Infections
Dolnośląskie	Moderately affected	603	0.17% (1/603)	0.17% (95% CI: 0.00–0.92%)	M; 47 y.o.; U
Kujawsko-pomorskie	Less affected	589	0.00% (0/589)	0.00% (95% CI: 0.00–0.62%)	0
Lubelskie	Moderately affected	597	0.00% (0/597)	0.00% (95% CI: 0.00–0.62%)	0
Łódzkie	Moderately affected	599	0.00% (0/599)	0.00% (95% CI: 0.00–0.61%)	0
Małopolskie	Moderately affected	605	0.17% (1/605)	0.17% (95% CI: 0.00–0.92%)	F; 24 y.o.; U
Podkarpackie	Less affected	619	0.16% (1/619)	0.16% (95% CI: 0.00–0.90%)	M; 39 y.o.; U
Podlaskie	Highly affected	698	0.29% (2/698)	0.29% (95% CI: 0.03–1.03%)	F; 45 y.o.; U F; 52 y.o.; U
Pomorskie	Less affected	598	0.33% (2/598)	0.33% (95% CI: 0.04–1.20%)	M; 47 y.o.; U M; 48 y.o.; R
Zachodniopomorskie	Less affected	599	0.00% (0/599)	0.00% (95% CI: 0.00–0.61%)	0
Overall		5507	0.13% (7/5507)	0.13% (95% CI: 0.05–0.26%)	

Abbreviations: CI = Confidence Interval, F = female; M = male; U = urban; R = rural.

**Table 2:** Distribution of post-vaccine immunity by voivodeships and characteristics of donors.

Characterization			% of VACCINATED (N)	Blood-Donors Characteristics -with Post-Vaccination Immunity					
Vodeship	Classification	Tested with Confirmatory Tests (N)		Female	Male	Younger (≤40 y.o.)	Older (>40 y.o.)	Urban	Rural
Dolnośląskie	M	603	1.7% (10)	4	6	5	5	8	2
Kujawsko-pomorskie	L	589	2.6% (15)	3	12	9	6	11	4
Lubelskie	M	597	3.2% (19)	6	13	12	7	8	11
Łódzkie	M	599	1.7% (10)	0	10	7	3	2	8
Małopolskie	M	605	2.5% (15)	3	12	11	4	9	6
Podkarpackie	L	619	1.9% (12)	1	11	2	10	5	7
Podlaskie	H	698	7.4% (52)	16	36	19	33	38	14
Pomorskie	L	598	2.8% (17)	3	14	11	6	12	5
Zachodnio-pomorskie	L	599	3.3% (20)	9	11	13	7	11	9
in total	5507		3.1% (170)	45/170 (26.5%)	125/170 (73.5%)	89/170 (52.4%)	81/170 (47.6%)	104/170 (61.2%)	66/170 (38.8%)

**Table 3:** Statistical analysis of aggregated data by classification of regions.

Classification	Reactivity % (N Reactive/N Tested)	*p*-Value for Reactivity	Seroprevalence % (N/N)	Exact 95% CIfor Seroprevalence	*p*-Value for Seroprevalence	Post-Vaccine Reactivity % (N/N)	Exact 95% CI for Post-Vaccine Reactivity	*p*-Value for Post-Vaccine Reactivity
Highly affected	9.6% (67/698)	<0.001	0.29% (2/698)	0.29% (0.03–1.03)	0.354	7.4% (52/698)	7.45% (5.61–9.65)	<0.001
Moderately affected	3.1% (75/2404)		0.08% (2/2404)	0.08% (0.01–0.30)		2.2% (54/2404)	2.25% (1.69–2.92)	
Less affected	5.4% (130/2405)		0.12% (3/2405)	0.12% (0.03–0.36)		2.7% (64/2405)	2.66% (2.06–3.39)	

Abbreviations: Reactivity = positive and bordeline results of screening ELISA IgG; seroprevalence = positive anti-NS1 TBEV ELISA result; post-vaccine reactivity = only neutralising Ab (VNT or IIFA positive result but anti-NS1 TBEV negative); CI = Confidence Interval, OR = Odds Ratio; *p* = *p* value, significant if *p* < 0.05.

## Data Availability

The original contributions presented in this study are included in the article. Further inquiries can be directed to the corresponding author.

## Abbreviations

The following abbreviations are used in this manuscript:

TBEV	Tick-borne encephalitis virus
TBE	Tick-borne encephalitis
VNT	virus neutralization testing
WHO	World Health Organization
RBTCs	Regional Blood Transfusion Centers
IIF	indirect immunofluorescence
ELISAs	enzyme-linked immunosorbent assays
IIFT	Indirect immunofluorescence test

## Appendix A

**Table A1:** The age groups of the donors involved in the project ‘TBE seroprevalence in Poland’.

Voivodship	Women		Men		Place of Living	
	≤40 y.o.	>40 y.o.	≤40 y.o.	>40 y.o.	Rural	Urban
Dolnośląskie	150	128	164	163	131	474
Kujawsko-pomorskie	49	82	273	196	214	386
Lubelskie	159	112	155	174	290	310
Łódzkie	170	123	174	133	271	329
Małopolskie	159	148	152	150	338	271
Podkarpackie	201	110	111	202	363	261
Podlaskie	173	130	211	189	161	542
Pomorskie	101	75	198	226	253	347
Zachodniopomorskie	150	150	150	150	148	452

**Table A2:** Epidemiological situation of TBE in Poland in years 2017–2022, based on data reported to surveillance system. Underestimation of reported symptomatic TBE cases based on the ratio Surveillance data/General Hospital Morbidity Research data [39].

Voivodeship	Our Classification Based on the Average TBE Incidence in 2015–2019	Average Incidence in 2017–2022	Underestimation (Fold) [Ratio Surv/Hosp 2017/2022]
Dolnośląskie	Moderately affected	0.88	0 [1.02]
Kujawsko-pomorskie	Less affected	0.04	2 [0.5]
Lubelskie	Moderately	0.71	1.1 [0.88]
Łódzkie	Moderately	0.34	1.22 [0.83]
Małopolskie	Moderately	0.55	1.25 [0.8]
Podkarpackie	Less affected	0.12	5.56 [0.18]
Podlaskie	Highly affected	8.24	1.41 [0.71]
Pomorskie	Less affected	0.07	2.56 [0.39]
Zachodniopomorskie	Moderately	0.14	1.22 [0.82]
Poland		0.68	1.32 [0.76]

**Table A3:** Distribution of reactive and seropositive samples by voivodeships.

Voivodeship	Classification Based on 5-Years TBE Incidence/ 100,000 Popul	Reactivity % (N/N)	Seropositivity % (N/N)
Dolnośląskie	Moderately affect	2.0% (12/605)	1.6% (11/603)
Kujawsko-pomorskie	Less affected	8.2% (49/600)	2.5% (15/589)
Lubelskie	Moderately affect	4.2% (25/600)	3.2% (19/597)
Łódzkie	Moderately affect	2.0% (12/600)	1.7% (16/605)
Małopolskie	Moderately affect	4.3% (26/609)	2.6% (16/605)
Podkarpackie	Less affected	3.5% (22/624)	2.1% (13/619)
Podlaskie	Highly affected	9.5% (67/703)	7.7% (54/698)
Pomorskie	Less affected	4.5% (27/600)	3.0% (18/598)
Zachodnio-pomorskie	Less affected	5.3% (32/600)	3.5% (21/599)
In total		4.9% (272/5541)	3.2% (177/5507)
